# Original papers: clinical or basic research anti-cyclic citrullinated peptide antibodies - activity markers in rheumatoid arthritis

**Published:** 2009

**Authors:** Denisa Predeteanu, Luminita Varzaru, Andra Balanescu, Violeta Bojinca, Daniela Opris, Violeta Vlad, Florian Berghea, Mihai Abobului, Cosmin Constantinescu, Ruxandra Ionescu

**Affiliations:** * Department of Internal Medicine and Rheumatology “Sf. Maria” Research Center on Pathology and Treatment of Rheumatic Diseases “Carol Davila”University of Medicine and Pharmacy, Bucharest, Romania

**Keywords:** anti-cyclic cytrullinated peptide antibodies, rheumatoid factor, rheumatoid arthritis

## Abstract

**Background**: *Immunological abnormalities in rheumatoid arthritis (RA) imply several antibodies, among which anti-cyclic cytrullinated peptide antibodies (anti-CCP) have the highest sensitivity and specificity. Their diagnostic and prognostic value in RA is well known, although their value as markers of the disease activity has not been established yet*.

**Objectives**: *The aim of this study is to evaluate the correlation between anti-CCP antibodies and RA activity which eventually leads to the best treatment of choice*.

**Patients and Methods**: *217 consecutive patients hospitalized in the Department of Internal Medicine and Rheumatology, “Sf. Maria” Clinical Hospital between 01.01-31.06 2007 were retrospectively studied. They were divided into two groups: group A-111 patients with RA (ACR criteria fulfilled) and group B-106 patients with other rheumatic diseases. The following parameters taken out of the patients, files were studied: parameters of the clinical activity of disease (C reactive protein, fibrinogen), rheumatoid factor (RF) and anti-CCP antibodies. Disease activity score (DAS) using 4 variables (number of tender joints, number of swollen joints, erythrocyte sedimentation rate and assessement of the disease activity) was also studied*.

*Data were processed with SPSS program using linear functions, Pearson correlation coefficient and Hi2 test of interdependency*.

**Results**: *The sensitivity of anti-CCP antibodies in patients with RA was **56.75%**. The specificity of anti-CCP antibodies in patients with RA was **90.56%**. Low seric levels of anti-CCP antibodies were also found in patients without RA, but with other conditions like: osteoarthritis, viral polyarthritis, infectious myositis and Still disease; moderate to high seric levels were found in patients with psoriatic arthritis*.

*Significant correlations were found between anti-CCP antibodies and DAS (r=0.437), between anti-CCP and fibrinogen (r=0,32) between anti-CCP antibodies and C reactive protein (r=0,237) as well as between anti-CCP and RF (r=0, 38)*.

**Conclusions**: *Anti-CCP antibodies are highly specific but moderately sensitive for RA, their highest frequencies and seric levels being found in seropositive RA. Anti-CCP can be used in patients with RA not only as a diagnostic marker but also as a reliable test for assessing the activity of the disease*.

## Introduction

Rheumatoid arthritis (RA) is an autoimmune disease, but the role played by the autoimmune reactions in the pathogenesis of RA has not yet been fully understood.

Early diagnosis of RA is extremely important as it allows remissive therapy to be initiated as soon as the disease sets on, thus avoiding its evolution to irreversible joint damage involved in disability and handicap of the patients. At the same time, an early diagnosis is difficult to be made in clinical practice, as it is based on symtomatology, and, the clinical picture at the onset of the disease is incomplete and often unspecific. Therefore, the identification of a specific antibody should be used as a serological marker in the early stages of the disease.[**1**,**2**]

Many autoantibodies, present at the onset of the disease, or even before it, have been described to be of diagnostic value. They are either specific to or associated with RA, the latter being present in other diseases too. They may be involved in the pathogenesis of RA initiating, maintaining and modulating the immune process. The rheumatoid factor (RF) is the most common of all nonspecific autoantibodies for RA. It has been the only serological criterion approved by the American College of Rheumatologists until present and, at the same time; it is considered an accurate criterion of classification for RA. 

Although these antibodies may have high sensitivity, their diagnostic value is lowered due to their low specificity and their presence in several other conditions; thus the number of autoantibody diagnosis tests should be increased.

 Among specific autoantibodies for RA, anti-cyclic citrullinated peptides (CCP) antibodies are used more often than others. They are very important for the diagnosis of the disease. It is well known that their specificity for RA is very high, although their sensitivity is similar to that of rheumatoid factor (RF). The anti-CCP antibodies have recently been set as a prognostic factor for RA, because patients with high levels of these autoantiobodies have distructive and erosive forms of the disease.[**4**,**5**]

In addition, the presence of anti-CCP antibodies can be detected long before the onset of clinical manifestations (14 years) which suggests their role in the pathogenesis of RA. Therefore, the presence of anti-CCP antibodies in patients with early RA will influence the therapeutic choice and optimal time to start the treatment.[**6**,**7**,**8**,**9**]

The aim of the study is to identify a correlation between the presence and the level of anti-CCP antibodies and RA clinical and biological activity. 

## Patients and methods

**FIG. 1:**
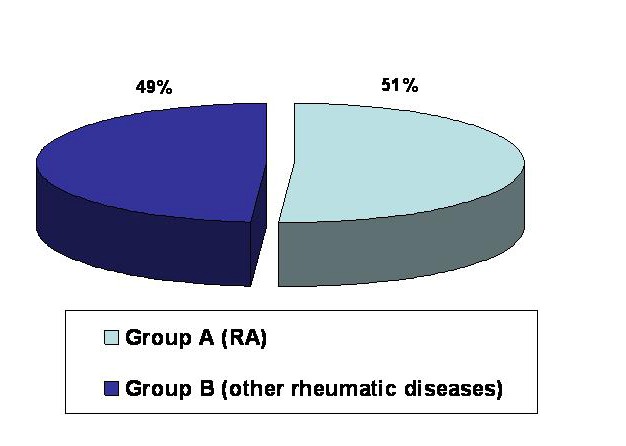
Study group distribution

The study was carried out retrospectively on **217** patients admitted to the Department of Internal Medicine and Rheumatology, “Sf. Maria” Clinical Hospital, Bucharest, Romania, between 01.01-31.06 2007. The patients were divided into two groups: group A-**111**(51%) patients fulfilling ACR criteria for RA and group B-**106** (49%) patients with undifferentiated arthritis whose further investigations upon the cases led to various diagnoses others than RA (**Fig. 1**)

**FIG. 2:**
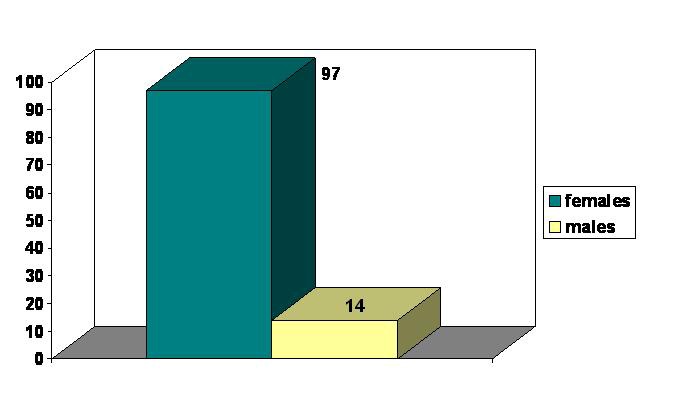
Sex distribution - group A

**FIG. 3:**
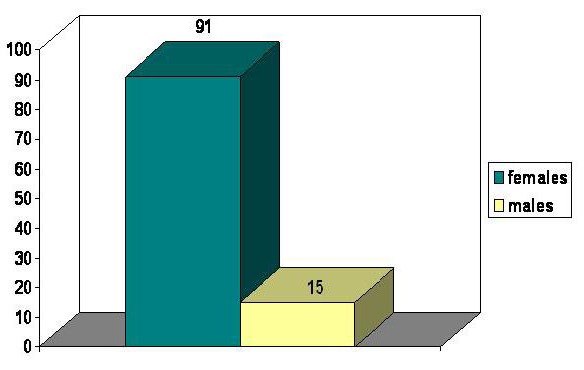
Sex distribution – group B

Demographic data were similar in both groups. All data were obtained from the patients’ observation sheets. The main age limits were 54.61┼-12.31 years in group A and 51.70┼-14.11 years in group B. Females/males ratio was 6.92 in group A and 6.06 in group B. (**Fig. 2**, **3**)

The study focussed on the following parametres:

-RA clinical and biological activity: DAS, C reactive protein, fibrinogen

-Presence of RF and its seric level

-Presence of anti-CCP antibodies and their seric levels

Statistical processing of data was performed with SPSS program using linear functions, Pearson correlation coefficient and Hi2 test of interdependency.

## Results

**FIG. 4:**
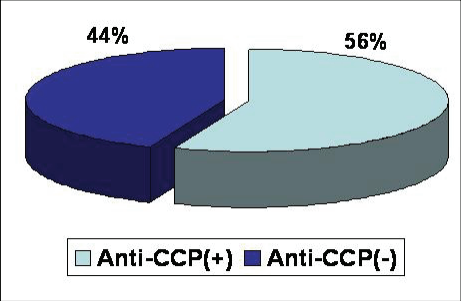
Anti-CCP antibodies sensitivity in RA

**FIG. 5:**
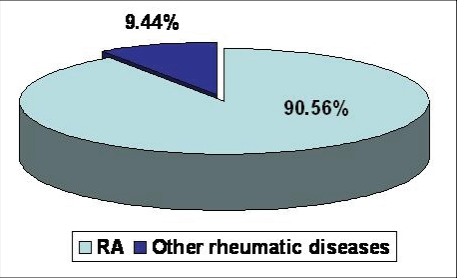
Anti-CCP antibodies specificity in RA

Before looking for a correlation between anti-CCP antibodies and the clinical and biological activity of RA we studied the sensitivity and specificity of these antibodies for RA. Anti-CCP antibodies tests using second generation kits are routinely performed in our clinic both for the early diagnosis and the follow-up of the disease. The tests for anti-CCP antibodies demonstrated sensitivity of **55.85%** (**Fig. 4**) and specificity of **90.56%** (**Fig. 5**)

Although anti-CCP antibodies specificity in RA is very high, small percentages of these antibodies have also been identified in other pathological conditions like: osteoarthritis, infectious myositis, viral polyarthritis, Still disease (slightly positive titres), systemic lupus erythematosus, psoriatic arthritis (moderately-intensely positive titres)

The present study evaluated the relationship between anti-CCP antibodies and the parameters which define the activity of RA, with regard to the correlation between anti-CCP antibodies and RA clinical and biological activity.

Thus, statistically, a very significant correlation was found between anti-CCP antibodies and DAS at the level of the whole group with RA.

To go even deeper into the matter, the distribution of patients was made according to DAS into: **inactive ** (DAS<2.6), **minimally active**(2.6<DAS<3.2), **moderately active** (3.2<DAS<5.1) and **intensely active** ( DAS>5.1) forms of the disease and according to the levels of anti-CCP antibodies into: **absent** (anti-CCP<20UI/ml), **slightly positive** (20<anti CCP<40 UI/ml), **moderately positive **(40<anti-CCP<80 UI/ml) and **intensely positive** (anti-CCP>80 UI/ml)

Statistically, there was a very relevant correlation between anti-CCP antibodies and DAS (r=0,437)

**FIG. 6:**
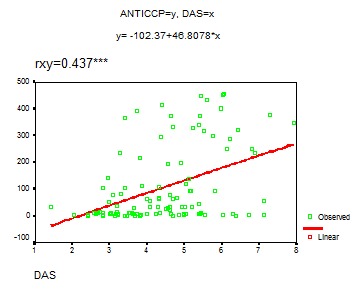
Anti-CCP-DAS Correlation

The patients with intensely active RA (DAS>5.1) have the highest level of anti-CCP antibodies (anti-CCP>80 UI/ml). The correlation between these two parameters -anti-CCP antibodies and DAS - persists only in the patients with moderately-intensely active RA, respectively with moderate-high titres of anti-CCP antibodies

**FIG. 7:**
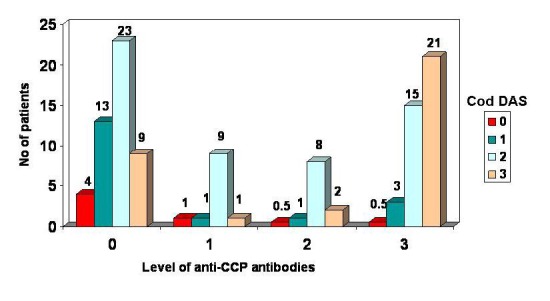
Distribuition of patients acoording to the anti-CCP level and DAS grade

The other parameters defining the clinical and biological activity of RA are C reactive protein and fibrinogen. Statistical analysis revealed significantly distinct correlations between anti-CCP antibodies and these two parameters (r=0.237 respectively r=0.32)

**FIG. 8:**
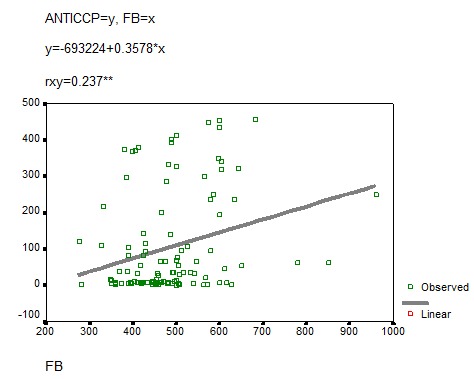
Anti-CCP-fibrinogen correlation

**FIG. 9:**
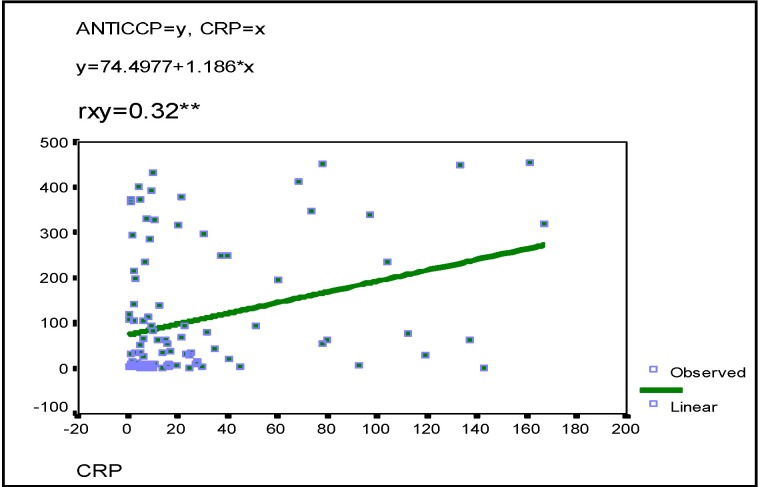
Anti-CCP-C reactive protein correlation

**FIG. 10:**
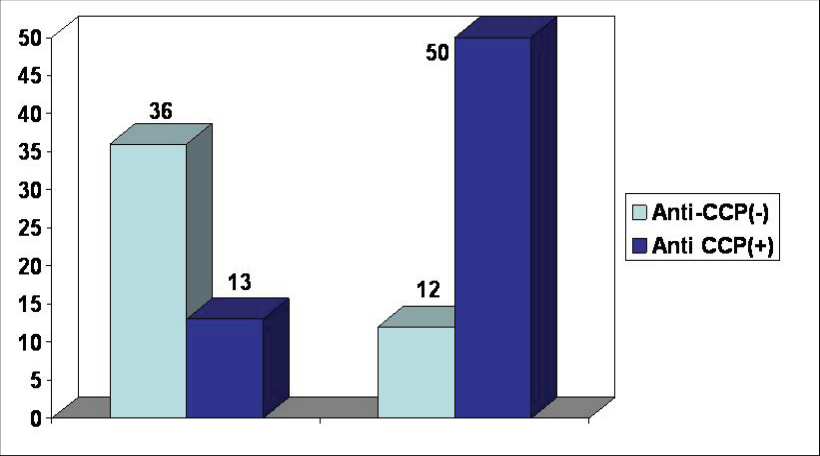
RA patients distribution according to the presence of anti-CCP and RF

In the group A of patients with RA, **62** (55,8%) were anti-CCP positive and **49** (44,2%) were anti-CCP negative, while **83** (74%) were seropositive (RF positive) and **28** (26%) were seronegative (RF negative). In the subgroup of patients with RA and anti-CCP positive, **50** (80, 6% ) were seropositive (RF positive) and **12** (19, 4%) were seronegative ( RF negative). In the subgroup of patients with RA and anti-CCP negative **13** (20, 4%) were seropositive (RF positive) and **36** (79, 6%) were seronegative (RF positive). This observation confirms that patients with RA and positive RF are mainly anti-CCP positive and patients with RA and negative RF are mainly anti-CCP negative (**Fig. 10**). 

An analysis of patients with RA according to the presence or absence of RF and the level of anti-CCP antibodies (0=, 1=, 2=, 3=) shows that patients with RF have the highest level of anti-CCP antibodies. 

Thus, in the subgroup of patients with seropositive RA (RF positive), 37 patients have the highest titre of anti-CCP antibodies. Compared to the subgroup of patients with RA seronegative (RF negative), only 2 patients have the highest titre of anti-CCP antibodies.

**FIG. 11:**
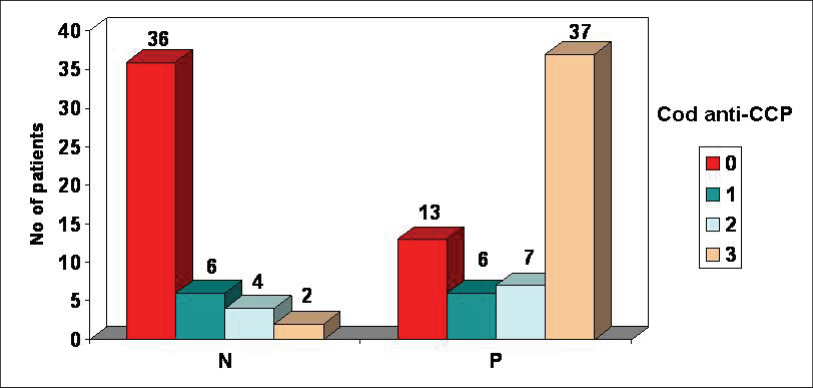
Patients distribution according to RF presence and anti-CCP antibodies level

The statistical analysis of the correlation between anti-CCP and RF in the group of patients with RA shows a **very relevant statistical correlation** (r=0,38)

**FIG. 12:**
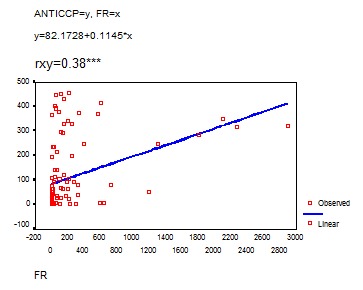
Anti-CCP and RF correlation

## Discussions

The specificity of anti-CCP antibodies in our study was **90.56%** and the sensitivity was **56.75%**, as compared to medical literature where the specificiy of anti-CCP is 98% and the sensitiviy is 80%.

The difference between the anti-CPP antibodies sensitivity according to our study and the data in literature seemed to be accounted for by the absence of anti-CCP in RA, possibly because the disease was inactive both clinically and biologically in some of the patients.

The present study considered anti-CCP antibodies to be positive at titres>20 UI/ml, since the threshold value of the seric level, suggesting the presence/absence of anti-CCP antibodies, has not been internationally standardized. Possibly, had a lower threshold value been used, the sensitivity would have been higher.

The high specificity of anti-CCP antibodies for RA has caused the results of the present study to be similar to the data in literature. The high specificity of these antibodies in patients with RA is explained by the local action of peptidylarginine deiminases (PAD) on peptide residues rich in arginine inducing citrullination.There are at least 5 genetically different forms of PAD, of which two isotypes, PAD2 and PAD4, have been found in monocytes and macrophages of inflamed synovium.[**10**,**11**] It was assumed that during rheumatic synovitis, PAD is released into the extracellular space inducing locally citrullination of arginine residues in several proteins (vimentin, fibrin and fibrinogen, fibronectin, keratin).[**12**] Therefore, anti-CCP antbodies have recently been suggested as being a new serologic criterion among ACR criteria for classification of RA.

In our study there was a statistically significant correlation between anti-CCP antibodies and serological markers of the activity of the disease (fibrinogen, C reactive protein). Moreover, there was a good correlation between anti-CCP antibodies and DAS, which is a very useful instrument in clinical practice for measuring the activity of RA.

The correlation between anti-CCP antibodies and the activity of RA is more obvious for moderately increased titres as well as for intensely increased titres. Thus, it is very important for clinicians to consider the initiation of a rapid and agressive treatment of the active forms of RA with moderately or intensely increased titres of anti-CCP antibodies.

## Conclusions

**1.** Anti-CCP antibodies proved to be a very important diagnostic test for patients with RA.

**2.** The specificity of this test was **90.56%** (similar to the results published by other authors) and the sensitivity was **56.75%** (lower than the results published by other authors).

**3.** Anti-CCP antibodies specificity was higher than that of RF, but the sensitivity was similar to that of RF. 

**4.** Anti-CCP antibodies are markers of RA activity due to their statistically significant correlation with DAS, fibrinogen and C reactive protein.

**5.** The correlation between anti-CCP antibodies and activity of RA was greater for moderately and highly increased titers of anti-CCP antibodies.

**6.** Seropositive forms of RA had the highest seric titres of anti-CCP antibodies. 

**7.** Identifying the anti-CCP antibodies in patients with RA is very useful in clinical practice not only as a diagnostic tool but also as a predictor of clinical outcome. 

**8.** The presence of the levels of anti-CCP antibodies may be an available test in order to choose the suitable treatment for the patient with rheumatoid arthritis.

The authors consider that the problems presented in this study may be very useful for practitioners when dealing with patients with RA, taking into account the diagnostic and prognostic value of anti-CCP antibodies. Therefore, further clinical studies must be made so as to highlight the role of anti-CCP antibodies in RA.
